# Under Concurrent Drought and Herbivory, Drought Dominates Herbivory on Morpho‐Physiological Responses in Soybean

**DOI:** 10.1111/ppl.70748

**Published:** 2026-01-25

**Authors:** Manish Gautam, Adriana Peissel, Rupesh Kariyat

**Affiliations:** ^1^ Department of Entomology and Plant Pathology University of Arkansas Fayetteville Arkansas USA; ^2^ Department of Crop, Soil, and Environmental Sciences University of Arkansas Fayetteville Arkansas USA

**Keywords:** abiotic stress, biotic stress, physiology, soil water content, tolerance

## Abstract

Drought and insect herbivory constantly threaten yield and productivity of crops like soybean ( *Glycine max* ). Recent advances in crop science have examined these stressors either individually or sequentially, but concurrent interactions have not been well understood. Therefore, using two soybean cultivars (Blackhawk‐ drought susceptible and Magellan‐ drought tolerant), we investigated how concurrent drought and herbivory by the fall armyworm ( *Spodoptera frugiperda* , FAW) affect soybean and FAW traits. Four treatments drought‐D, herbivory‐H, drought × herbivory‐DH, and well‐watered‐WW were imposed at the third‐trifoliate stage (V3) for a week. During the treatment period, daily measurements of net photosynthesis rate, stomatal conductance, transpiration rate, and soil moisture were taken, whereas plant height and chlorophyll content were recorded during alternate days throughout the treatment period. In addition, FAW mass gain was also measured daily. Leaf trichomes, a major physical defense system in plants, were estimated immediately post treatment and six days after. Our results showed that concurrent DH significantly impaired physiological traits, reduced the soil water content, and affected plant growth. Trichomes were significantly higher under DH compared to WW and the effect persisted after treatment. Although FAW performed similarly in both drought‐stressed and well‐watered plants, strong cultivar effects were observed for larval mass gain and FAW seemed to perform better on the drought susceptible cultivar. This study established a clear trend showing drought is the dominant stressor on soybeans, compared to herbivory alone and hence informs the growers for prioritization of stress management. Overall, this study presents novel insights into the effects of concurrent drought and herbivory, with implications for resistance breeding using tolerant cultivars against stressors like herbivory and drought.

## 1 Introduction

Agricultural crops are under constant pressure due to dual stressors like drought and herbivory, particularly in arid and semi‐arid regions where prolonged drought stress is becoming increasingly common. Continuously changing climatic conditions can further aggravate the interactive effects of drought and insect herbivory worldwide (Grinnan et al. 2013a; Peschiutta et al. 2016; Skendžić et al. 2021). While most of the current studies have attempted to explore their interactive effects in agricultural crops, the complexity and the multi‐faceted aspects of the interactions have been proven to be far more challenging. Recent meta‐analysis suggests that drought and herbivory interact and impact crop physiology and phytohormones (Gautam and Kariyat 2025a), and can have prominent effects on crop morpho‐physiology and yield parameters when they occur in a sequence (Shafi et al. 2024). For example, Gautam et al. (2024) reported that when soybean plants were infested by soybean looper (*Chrysodeixis includens*, SBL) after a week of drought stress, the plants compensated physiologically in a strengthened defense but all this resulted in poor yields. In contrast, Ayala et al. (2025) reported that there were no significant effects of drought by itself or when combined with SBL and fall armyworm (*Spodoptera frugiperda*, FAW) on soybean physiological traits. However, a crucial factor potentially affecting these discrepancies in results is the use of several genotypes which differ in their water use efficiency (WUE) and their tolerance towards limited water availability and herbivory (Ayala et al. 2025).

Use of tolerant and resistant genotypes against drought and/or herbivory, respectively, could eventually lead to the development of climate resilient crops (Rasheed et al. 2022). In a recent study, a drought tolerant cultivar of soybean, Magellan, was found to have a considerably stable performance in terms of morpho‐physiological parameters compared to the drought susceptible cultivar Blackhawk (Gautam et al. 2024). Specifically, Magellan had better adaptability in terms of root growth and physiological traits such as net assimilation rate and stomatal conductance under drought conditions but was less affected by SBL infestation. On the contrary, Faustino et al. (2021) reported that both drought resistant and susceptible genotypes experienced a reduction in herbivory, and the use of contrasting genotypes did not affect the survival of velvetbean caterpillar ( *Anticarsia gemmatalis* ) under drought. Although genotypic variations can have significant effects on drought and herbivory interactions, drought seems to be more dominant as compared to herbivory alone. For example, Shibel and Heard (2016) reported that drought was found to be more dominant compared to simulated herbivory in two species of goldenrod. In another study, Grinnan et al. (2013b) found that drought had a significantly greater effect on soybean performance compared to herbivory (independent incidence). This is mainly because drought stress can cause catastrophic hydraulic failure in plants along with damage to the photosynthetic apparatus in the leaves (Klein et al. 2014)‐ far more lethal than herbivory. Therefore, both from a crop improvement and agroecology standpoint, there is an urgent need to understand how the host and herbivores perform under such abiotic and biotic stressors, specifically, drought and herbivory acting simultaneously.

Crop performance, in terms of physical and chemical defenses, is even less explored under drought and herbivory interactions. Faustino et al. (2021) found that abscisic acid (ABA) acted synergistically with jasmonic acid (JA) to induce more defense responses in soybean upon simultaneous drought and herbivory when compared to the independent stressors. Not only that, drought and herbivory have been independently found to impact leaf trichomes, which are one of the most important physical defenses in plants, including soybeans (Coapio et al. 2018; Gandham et al. 2024; Harish et al. 2023). In response to drought stress, wheat leaves have been found to induce trichomes on both the adaxial and abaxial side of the leaves (Farooq et al. 2009). Furthermore, recently we reported that sequential drought and herbivory stress can induce trichomes in the parents' generation (Gautam et al. 2026) as well as transgenerational plants which lead to strong trade‐offs between growth‐physiology‐yield in soybeans (Gautam and Kariyat 2025b). More importantly, drought tolerant genotypes have been found to possess higher trichome density than drought sensitive genotypes in soybeans (Wei jun et al. 2009). Undoubtedly, concurrent interactions of abiotic and biotic stressors like drought and herbivory play a significant role in driving the host and herbivore performance via induced crop phenotypes and a potential cascade of defense mechanisms. However, all of these studies tested either independent effects of drought and herbivory or when drought and herbivory occurred in a sequence. The concurrent effects of dual stress on host and herbivore performance in addition to the role of genotypic characteristics remain unexplored.

In this regard, a critical question is how drought interacts with a generalist versus a specialist herbivore and what are the consequences for the host and the herbivore? Previously, we found that SBL, which is more specialized on soybeans, was less attracted to drought stressed soybeans compared to well‐watered plants (Gautam et al. 2024). However, FAW, a generalist herbivore that can infest more than 370 host species (Qiu et al. 2020; Li et al. 2022; Balakrishnan et al. 2024), responds differently to such drought stressed conditions. This is mainly because drought stress‐mediated physiological changes in host plants also affect their nutritional status, and the feeding behavior of a specialist and generalist herbivore is largely associated with host nutritional values. For example, low nutritional content in the host diet significantly prolonged the larval development of FAW in cotton and soybean plants and FAW was found to least prefer these two host crops compared to corn, oat, and wheat (da Silva et al. 2017). Similarly, a close relative of FAW, the beet armyworm *Spodoptera exigua* , was found to feed more on drought stressed compared to well‐watered cotton leaves (Showler and Moran 2003). Likewise, in another study, the cotton leafworm *Spodoptera littoralis*, a generalist, was reported to prefer severely drought stressed garlic mustard plants (Gutbrodt et al. 2011). Therefore, it is possible for FAW to perform better when they feed on drought stressed soybean plants compared to SBL. Furthermore, despite being a less preferred host (Pitre et al. 1983), FAW can feed voraciously and successfully complete their lifecycle on soybeans (Ayala et al. 2024; Gandham et al. 2024). Owing to their highly polyphagous nature and ability to migrate up to thousands of kilometers annually (Nagoshi et al. 2023), FAW are now considered a critical concern to global agriculture. With increasing drought frequencies and rising global temperatures, FAW outbreaks can be a major concern moving forward for soybean growers across the world.

Taken together, we intended to explore how concurrent drought and herbivory affect host and herbivore performance using soybeans and FAW as our study systems. We performed a comprehensive examination of soybean morpho‐physiological traits (net photosynthesis rate, stomatal conductance, transpiration rate, and leaf chlorophyll content), plant height, and defenses (leaf trichomes). We also assessed the larval performance of FAW when they fed drought stressed soybeans throughout the entire stress period. We hypothesized that with increasing drought stress, soybean physiological traits will rapidly deteriorate, but FAW larvae will continuously feed and perform better as the degree of drought stress continues to rise. Additionally, we predicted that drought stress will be more dominant than herbivory stress and that drought susceptible cultivars will exhibit less stable physiology and will be preferred by FAW compared to the drought tolerant cultivars. By using cultivars of contrasting drought tolerance alongside a generalist herbivore like FAW, this study provides novel insights into the effects of concurrent drought and herbivory.

## 2 Materials and Methods

### 2.1 Study Systems

These experiments were conducted in the greenhouse located at the Milo J. Shult Agricultural Research & Extension Center (SAREC), Arkansas Agricultural Experiment Station, University of Arkansas. The study included two soybean cultivars: Blackhawk (drought‐sensitive; Sammons et al. 1978) and Magellan (drought‐tolerant; He 2008). The details of the cultivars are available in Table S1, adapted from Supporting Information of Gautam et al. (2024). The seeds were obtained from the previous season of plants grown in the same greenhouse. All the plants were grown in LB15 potting soil (Farmers Co‐Op, Van Buren), fertilized biweekly with Osmocote Plus 15‐9‐2 (ICL Specialty Fertilizers), and maintained under standard greenhouse conditions (16:8 light: dark cycle, ~70% relative humidity (RH), and a temperature range of 28°C–30°C). The plants were well‐watered until saturation, except during the drought treatment period.

The eggs of fall armyworms were bought from a commercial supplier (Frontier Agricultural Services) and reared in the laboratory at University of Arkansas. A wheat‐germ‐based artificial diet was used to rear the larvae (Tayal et al. 2020). The larvae were raised under laboratory conditions (25°C–28°C, ~70% relative humidity) until the 2nd instar and then used for the experiments.

### 2.2 Concurrent Drought and Herbivory Treatments

**FIGURE 1:**
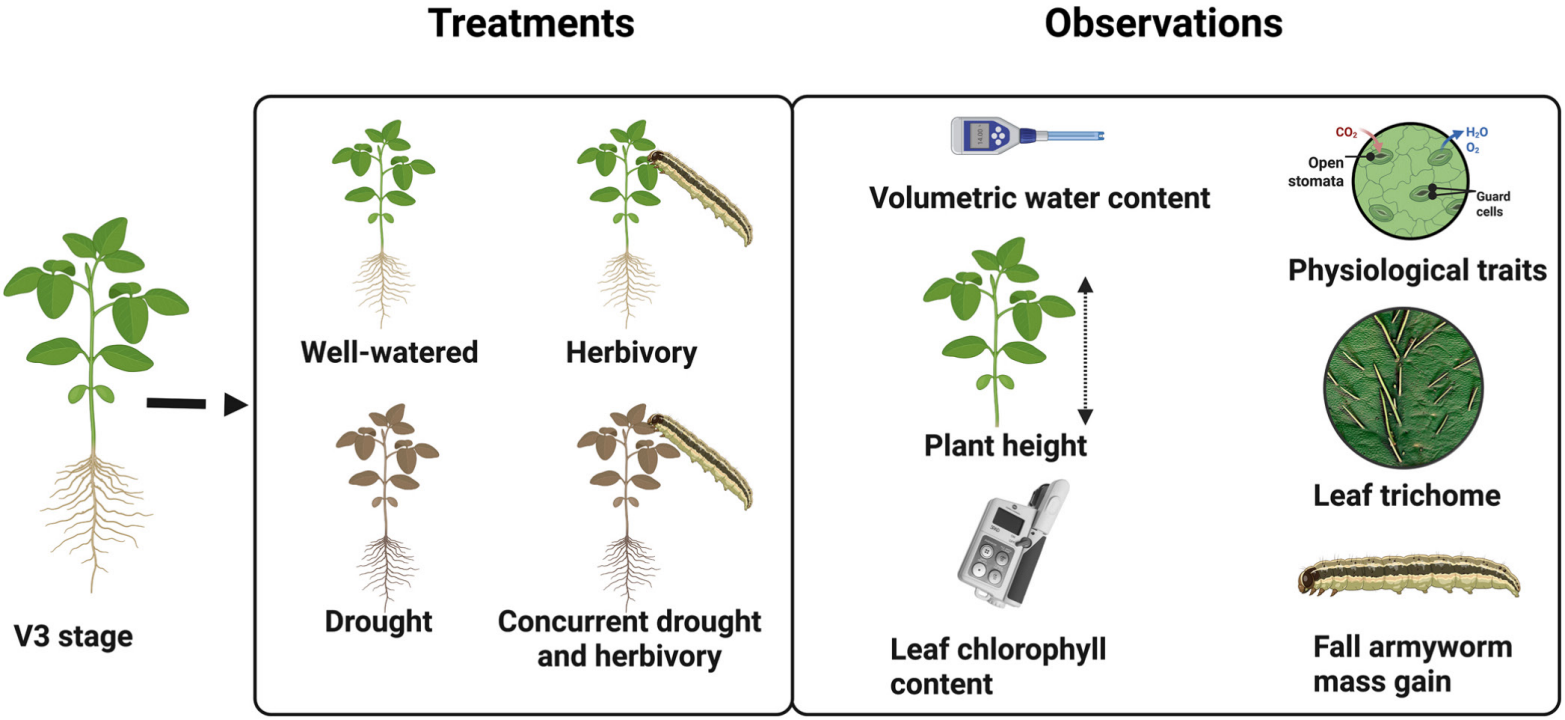
Graphical representation of concurrent drought and herbivory treatment and the observed parameters in this experiment. The treatments were imposed at third trifoliate stage (V3) soybean plants for 7 days. Under concurrent drought and herbivory treatment, the plants were under drought stress as well as fed by 3^rd^ instar fall armyworm caterpillars for the entire duration of the treatment period. The volumetric water content, physiological traits, and fall armyworm mass gain were measured daily while plant height and leaf chlorophyll content were recorded on alternate days. The leaf trichome density was assessed immediately after and 6 days after ending the treatment. The image is generated using Biorender.com.

Drought and herbivory treatments were imposed on soybean plants at the same time for one week. At stage V3 (third trifoliate stage), 160 plants (80 Magellan and 80 Blackhawk plants) were divided into four treatment groups (drought‐D, herbivory‐H, drought × herbivory‐DH, and well‐watered‐WW) with 20 plants per treatment per cultivar (40 plants per treatment). Plants under D and DH treatment were not watered for one week, while under H, FAW larvae (2nd instar) were allowed to feed on well‐watered plants for the entire week. As for the DH treatment, FAW was released in addition to the drought treatment, whereas WW treated plants were watered normally, and no stress was imposed (Figure 1). Soil moisture content was measured using a soil moisture probe (Bluelab metpulse pulse meter, Bluelab Corporation Limited) to assess the volumetric water content (%VWC) daily throughout the treatment period. The concurrent drought and herbivory treatment ended a week after the initiation of each treatment, and the plants were regularly watered while the FAW were removed. The plants were then allowed to grow normally and mature under normal conditions and regular management.

### 2.3 Data Collection and Observations

#### 2.3.1 Morpho‐Physiological Measurements

Plant height and leaf chlorophyll content were measured on alternative days during the treatment period. Leaf chlorophyll content was measured by using a SPAD 502 chlorophyll meter (Spectrum technologies Inc) from the middle leaflet of the youngest fully expanded trifoliate leaf on each plant. A portable photosynthesis system model LI‐6400/XT (LI‐COR Biosciences) was used for daily measurements of physiological parameters during the treatment period. For a detailed explanation of the settings for LI‐6400/XT, see Gautam et al. (2024). Physiological traits measured include net photosynthesis rate (μmmol CO_2_ m^−2^ s ^−1^), stomatal conductance (mol m^−2^ s^−1^), and transpiration rate (mmol H_2_O m^−2^ s ^−1^). The measurements were carried out every day between 10:00 am and 1:00 pm for consistency.

#### 2.3.2 Assessment of Leaf Trichomes

To understand the effect of concurrent drought and herbivory on leaf trichomes, leaves were collected from 10 plants from each treatment per cultivar (*n* = 20 per treatment). The leaves were excised at the petiole 0 days immediately after the treatment and six days after the treatment ended (a week later including the day of ending the treatment) and observed under a compound microscope (Carl Zeiss Axiolab RE, Germany) at 10× magnification (Balakrishnan et al. 2024; Gautam and Kariyat 2025b). The non‐glandular trichomes in the leaves were counted per 0.086 mm^2^ leaf area and trichome density was determined under each treatment (Figure S1). All the observations for leaf trichomes were performed by the same individual for consistency.

#### 2.3.3 FAW Mass Gain

Larval measurement (mass) was taken daily for each plant during the experimental period. FAW larvae were weighed initially before putting them on the plants (initial weight) and weighed every 24 h every day (final weight) throughout the treatment period (1 FAW per plant). The mass gain of the FAW was calculated based on the following equation (Ayala et al. 2024; Gautam et al. 2024).

$$\%\mathrm{FAW}\;\text{mass gain}=\frac{\text{Final weight}-\text{initial weight}}{\text{Initial weight}}\times 10$$

### 2.4 Statistical Analysis

The statistical model for analyzing the response variables included two main factors: the treatments and the cultivars. For continuous variables like volumetric water content, plant height, chlorophyll content, physiological parameters, and FAW mass gain, analysis of variance (ANOVA) was conducted followed by Turkey's HSD test. When the assumptions of ANOVA were not met, log or square root transformations were carried out. For the non‐parametric test, a Kruskal Wallis test was performed followed by a Dunn's test, and a generalized model with Poisson distribution was carried out for trichome count data. Data was processed using the data.table package (Dowle and Srinivasan 2023) for data wrangling, and figures were created using the ggplot2 package in R studio 2024.12.0 (R Core Team 2023). Full factorial ANOVA was carried out to test the effects on response variables based on treatments (D, DH, H, and WW), cultivars (Blackhawk and Magellan), and interactions between treatment and cultivar. A detailed statistical summary is provided in the Tables S2–S5, and the experimental design of this study is presented through the schematic diagram in Figure 1.

## 3 Results

### 3.1 Effects on Volumetric Soil Water Content

**FIGURE 2:**
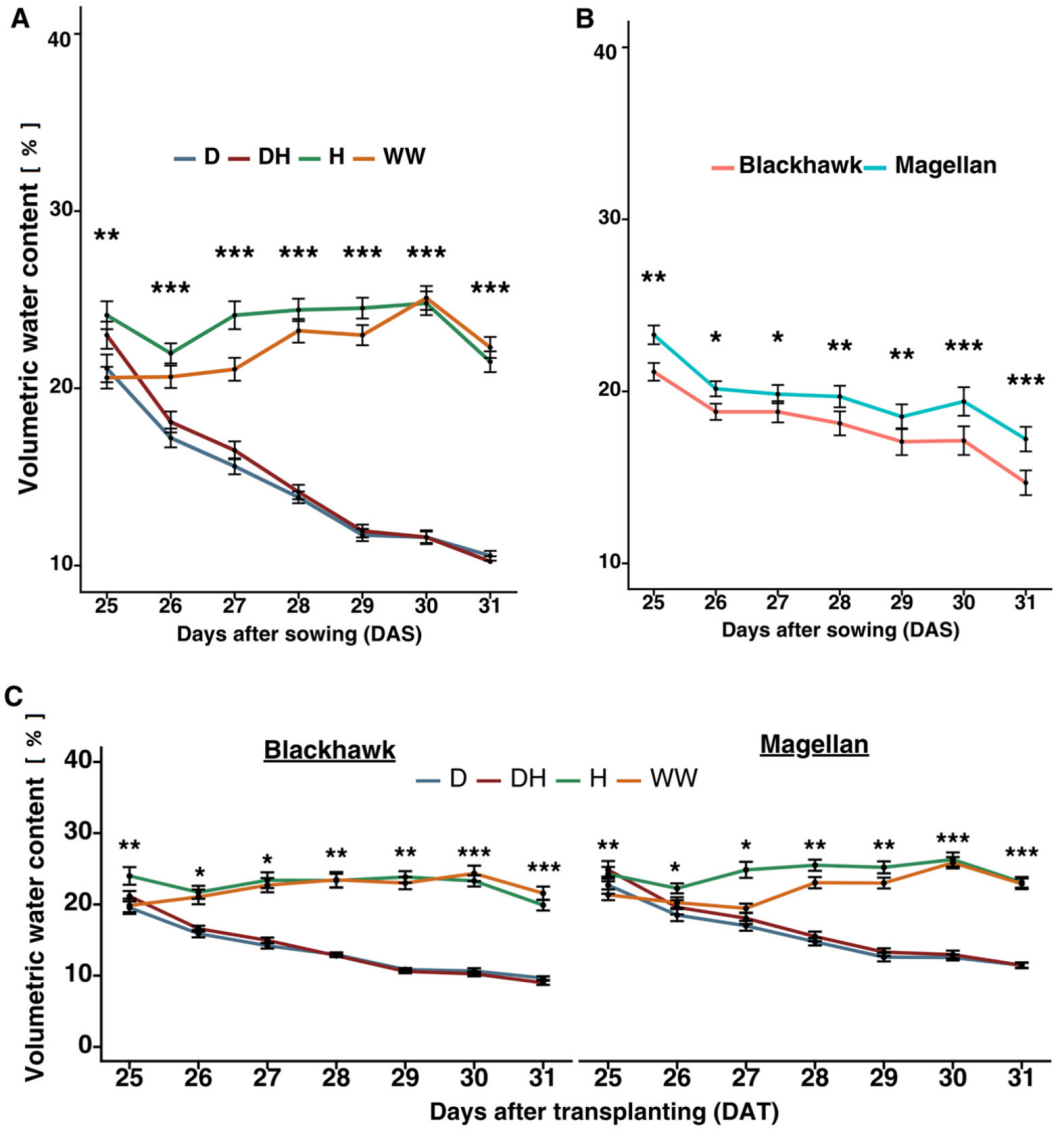
Volumetric soil water content (%) across treatments and cultivars over a drought and herbivory treatment period. (A) Soil moisture trend under drought and herbivory treatment effects, and (B) soil moisture trend for two cultivars (Blackhawk and Magellan). Drought and herbivory treatments are drought (D), drought × herbivory (DH), herbivory (H), and well‐watered (WW). *p < 0.05; **p < 0.01; ***p < 0.001; ns, not significant.

As expected, volumetric soil water content was significantly affected by drought and herbivory treatments across both soybean cultivars. On Day 25 (pre‐treatment), although a significant effect was observed, the volumetric water content had very small differences among the treatments (Figure 2A), confirming uniform starting conditions. However, on Day 26, the first day after the treatment, soil moisture declined rapidly in drought (D) and drought × herbivory (DH) treatments, while remaining stable in well‐watered (WW) and herbivory (H) treatments (df = 3, *p* < 0.001, Figure 2A). The average VWC for plants under H and WW treatments was around 20%–25%, whereas it was around 10%–12% for D and DH treated plants. Across the treatment period, when volumetric soil water content data was pooled from all treatments, Magellan was found to retain significantly more soil water content than Blackhawk (Figure 2B). Under the interactive effects of drought and herbivory treatments, Blackhawk lost water significantly faster than Magellan under water stress conditions (Figure 2C). This shows that Magellan is better at retaining water under stress conditions compared to Blackhawk and both D and DH treatments are distinctly different from H and WW in terms of volumetric soil water content.

### 3.2 Effects of Drought × Herbivory on Plant Height

**FIGURE 3:**
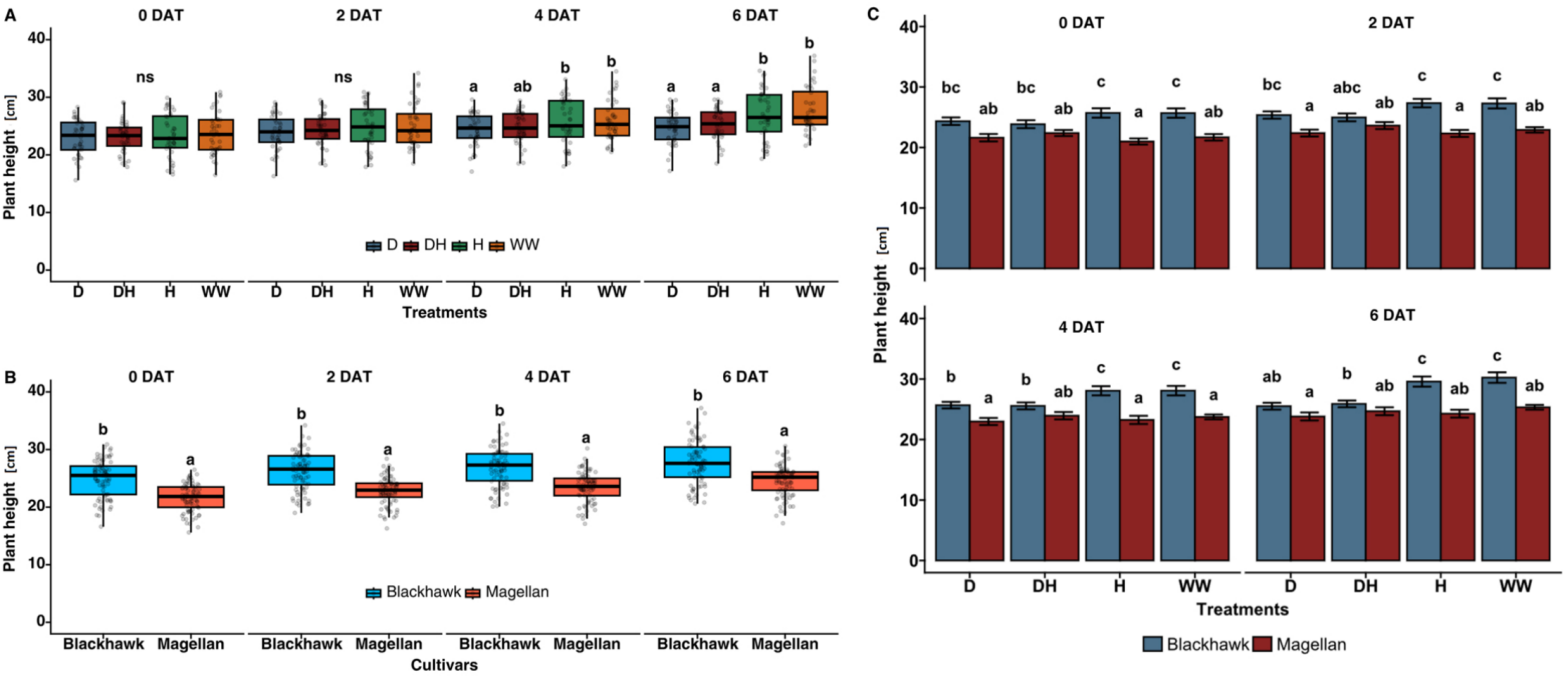
Effects of stress treatments and cultivars on plant height. (A) Plant height under drought and herbivory treatment effects, and (B) Plant height for two cultivars (Blackhawk and Magellan) at 0, 2, 4, and 6 days after treatment started (DAT). The height of the plants was measured every alternate day starting from the day the treatment was imposed. Drought and herbivory treatments are drought (D), drought × herbivory (DH), herbivory (H), and well‐watered (WW). Different letters in treatments indicate significant differences at the 5% level of significance, and data are presented as mean ± SE (standard error). ns, not significant.

Plant height was significantly affected by both drought and combined drought × herbivory treatments across the experimental period. Prior to the treatments, that is, 0 days after the treatment started (DAT; 0 DAT), there were no significant differences in height among the treatment groups (df = 3, *p* > 0.05). However, over the course of the study, plants under well‐watered (WW) and herbivory‐only (H) treatments showed significantly greater height compared to those under drought (D) and drought × herbivory (DH) treatments (df = 3, *p* < 0.001; Figure 3A). By the end of the treatment phase (6 DAT), plants in the DH group exhibited the shortest average height compared to H and WW treatments, indicating a compounding negative effect of combined abiotic and biotic stress. However, since DH was statistically similar to the D treatment, this effect seems to be largely driven by drought stress only. Cultivar type also had a significant effect on plant height (*p* < 0.01). Blackhawk exhibited greater changes in height (reduced under drought and DH treatments) but no change was observed in Magellan (df = 3, *p* < 0.05, Figure 3C).

### 3.3 Chlorophyll Content

**FIGURE 4:**
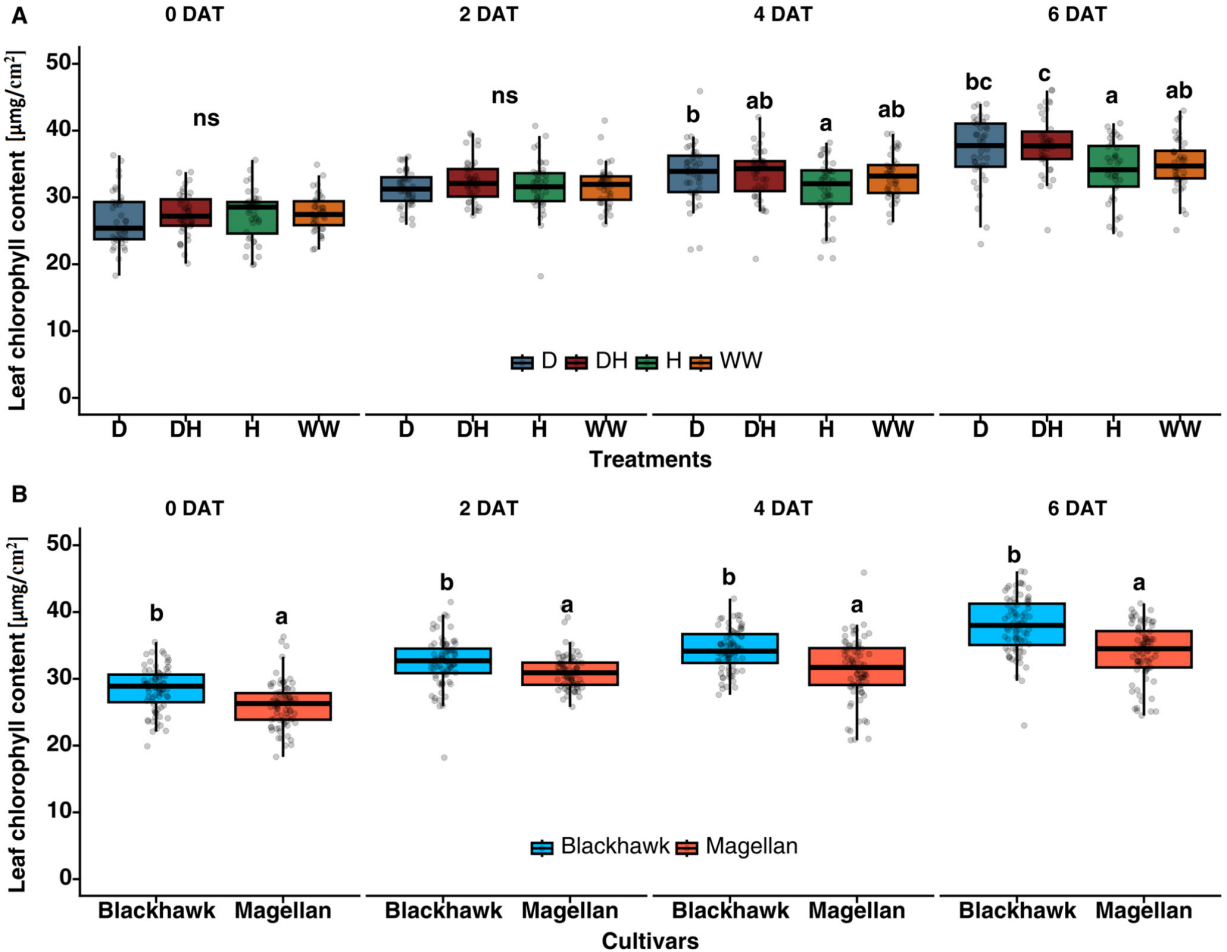
Effects of treatments and cultivars on leaf chlorophyll content. (A) Leaf chlorophyll content under drought and herbivory treatment effects, and (B) for two cultivars (Blackhawk and Magellan). The measurement was obtained every alternate day at 0, 2, 4, and 6 days after the treatments were imposed (DAT). Drought and herbivory treatments are drought (D), drought × herbivory (DH), herbivory (H), and well‐watered (WW). Different letters in treatments indicate significant differences at the 5% level of significance, and data are presented as mean ± SE (standard error). ns, not significant.

At the start of the treatment period (0 DAT), there were no significant differences in chlorophyll levels among the treatment groups (df = 3, *p* > 0.05) or between the cultivars (df = 1, *p* > 0.05). By 4 DAT and 6 DAT, plants under drought (D) and drought × herbivory (DH) treatments exhibited significantly higher chlorophyll levels than those under well‐watered (WW) and herbivory (H) conditions (df = 3, *p* < 0.01, Figure 4A). These changes suggest a physiological response to drought and herbivory attack under stressed conditions. Also, Blackhawk exhibited significantly higher leaf chlorophyll content than Magellan throughout the treatment duration (df = 1, *p* < 0.05, Figure 4B). There were no significant interaction effects between treatment and cultivar (*p* > 0.05), indicating that both cultivars responded similarly to drought and herbivory treatments regarding chlorophyll retention.

### 3.4 Effects on Physiological Traits

**FIGURE 5:**
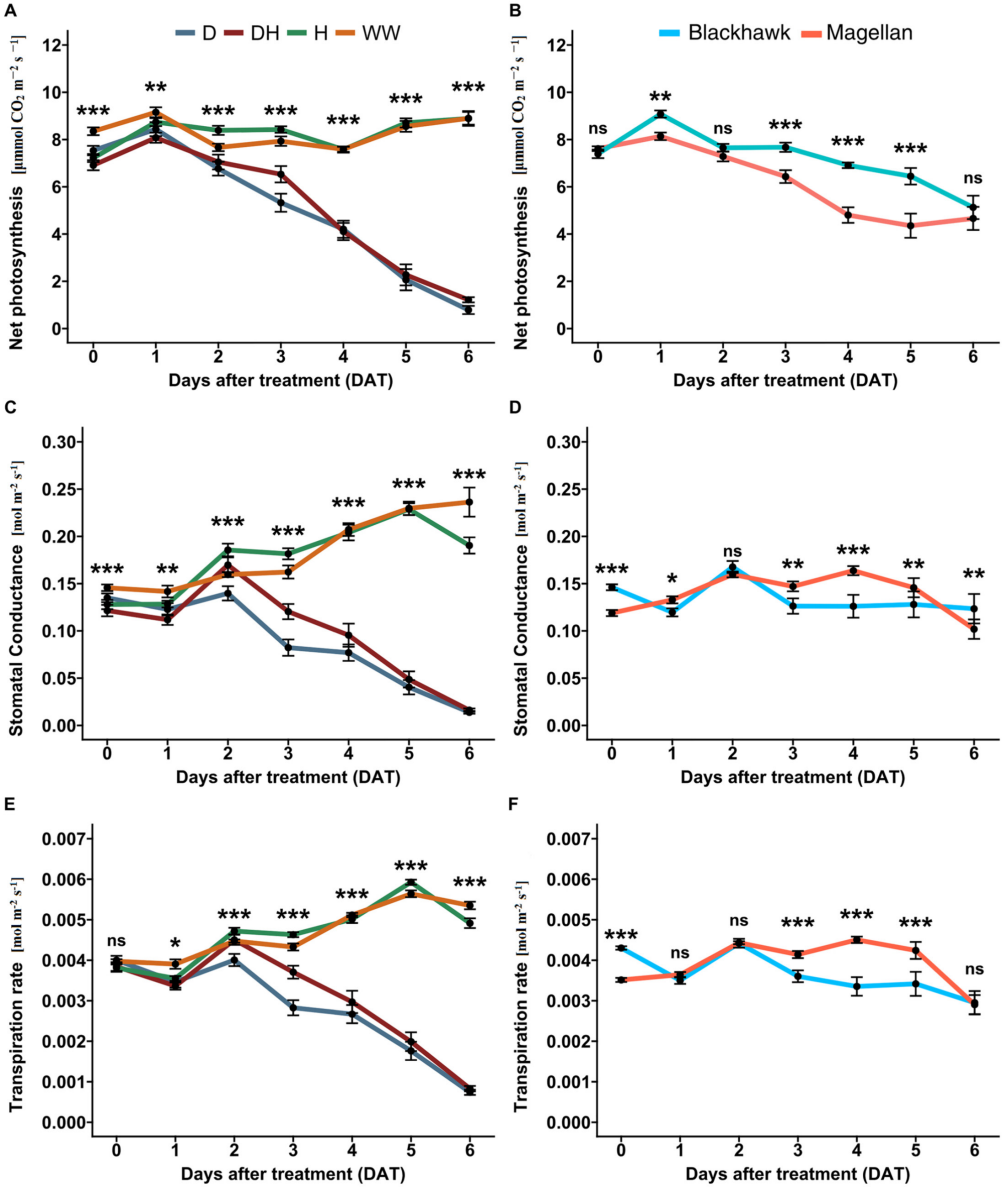
Physiological traits of soybeans under drought and herbivory treatments for two cultivars for each day throughout the treatment period. Net photosynthesis rate for each day during the drought and herbivory treatment period under the (A) effects of treatment, (B) and cultivars (Blackhawk and Magellan). Stomatal conductance for each day during the drought and herbivory treatment period under the (C) effects of treatment, and (D) cultivars (Blackhawk and Magellan). Transpiration rate for each day during the drought and herbivory treatment period under the (E) effects of treatment, and (F) cultivars (Blackhawk and Magellan). Drought and herbivory treatments are drought (D), drought × herbivory (DH), herbivory (H), and well‐watered (WW). * *p* < 0.05; **p < 0.01; ***p < 0.001; ns, not significant.

Treatments and cultivars had significant effects on soybean physiological traits. Net photosynthesis rate was significantly reduced in drought (D) and drought × herbivory (DH) treatments compared to well‐watered (WW) and herbivory (H) conditions (df = 3, *p* < 0.001; Figure 5A). For both WW and H treatments, the net photosynthesis rate ranged from 7 to 10 μmol CO_2_ m^−2^ s^−1^ throughout the treatment period but it rapidly declined under D and DH from an average of 7 μmol CO_2_ m^−2^ s^−1^ to as low as 1 μmol CO_2_ m^−2^ s^−1^ (Figure 5A). Compared to Blackhawk, Magellan had consistently higher photosynthetic rates throughout the treatment period (df = 1, *p* < 0.01; Figure 4B). Stomatal conductance followed a similar pattern, with significant reductions under D and DH treatments (df = 3, *p* < 0.001; Figure 5C), but Magellan had greater values than Blackhawk mostly during the later stages of the treatment period (Figure 4D). The transpiration rate also declined significantly under D and DH (df = 3, *p* < 0.001; Figure 4E) and the cultivar effects followed the trend observed for stomatal conductance. Magellan also exhibited greater transpiration during the later stages as the stress was prolonged longer. Overall, a rapid decline in both stomatal conductance and transpiration rate was observed under D and DH treatments which were almost five times lower than that of WW and H treatments.

The principal component analysis (PCA) of all physiological traits under drought and herbivory treatments showed that components 1 and 2 explained 98.2% of the variation in the data (Figure S2A). Well‐watered (WW) and herbivory (H) conditions are closer together, while drought (D) and drought × herbivory (DH) treatments spread out more, suggesting stronger physiological changes under drought. In the case of the cultivars, components 1 and 2 explained 95.1% variation in the data (Figure S2B). From this PCA, it was clear that the two cultivars exhibit distinct physiological responses. Furthermore, the treatments and the cultivars also interacted significantly and presented a clear trend in terms of physiological traits. Magellan exhibited more stable physiology under D and DH treatments, while Blackhawk showed a more rapid decline in net photosynthesis rate (Figure S3A), stomatal conductance (Figure S3B), and transpiration rate (Figure S3C). Also, towards the later stage of the treatment period, all the physiological traits were found to be significantly lower under D and DH treatments in Blackhawk compared to Magellan.

These results collectively indicate that soybean plants exhibit a pronounced physiological response under drought and drought × herbivory stress conditions, while herbivory had little impact on physiological traits. At the same time, cultivar effects strongly drove the physiological responses of the plants when pooled across the treatments as well as under the interactive effects with the drought and herbivory treatments.

### 3.5 Trichome Density Immediately After the Treatment Period

**FIGURE 6:**
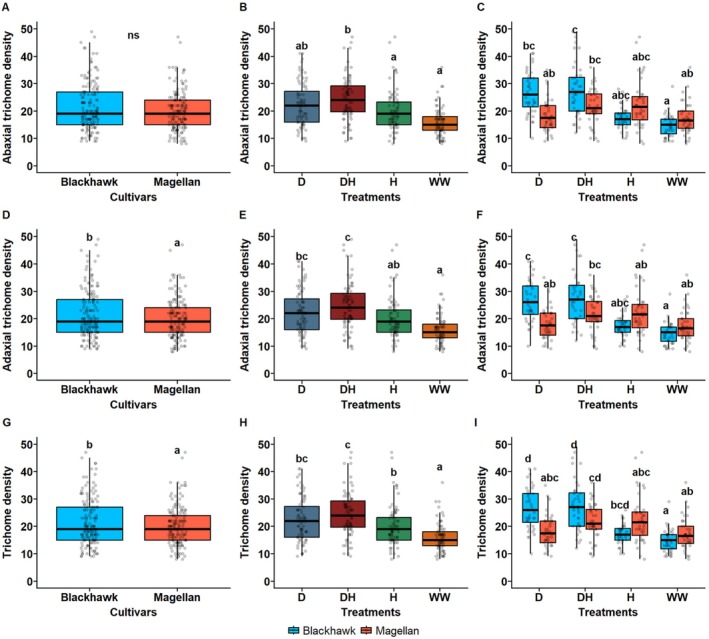
Effects of treatments and cultivars on leaf trichomes immediately after ending the treatment. (A) Adaxial trichome density for two cultivars, (B) under drought and herbivory treatment, and (C) under interactive effects of treatments and cultivars. (D) Abaxial trichome density for two cultivars, (E) under drought and herbivory treatment, and (F) under interactive effects of treatments and cultivars. (G) Average trichome density (pooled across adaxial and abaxial surface) for two cultivars, (H) under drought and herbivory treatment, and (I) under interactive effects of treatments and cultivars. The leaves were excised on the same day the treatment period ended. Leaf trichome density was measured by observing them under a compound microscope (0.086 mm^2^ area) under 10× magnification. Drought and herbivory treatments are drought (D), drought × herbivory (DH), herbivory (H), and well‐watered (WW). Different letters in treatments indicate significant differences at the 5% level of significance, and data are presented as mean ± SE (standard error). ns, not significant.

Both treatments and cultivars showed strong effects on leaf trichome density. Immediately following the ending of the treatment period, abaxial trichome density was statistically similar across both cultivars (df = 1, *p* > 0.005, Figure 6A), whereas plants exposed to drought × herbivory (DH) and drought (D) treatments had the highest abaxial trichome densities (df = 3, *p* < 0.001, Figure 6B) and well‐watered (WW) and herbivory (H) plants had the lowest. Similarly, interactive effects of the treatments and cultivars were also significant on the abaxial trichomes, with Blackhawk having the highest density of trichomes under D and DH treatments (df = 3, *p* < 0.001, Figure 6C).

Meanwhile, adaxial trichome density was significantly greater in Blackhawk compared to Magellan (df = 1, Figure 6D), but the effects of drought and herbivory treatment followed a similar trend seen in adaxial trichomes; DH treatment induced significantly higher trichomes in the adaxial surface as well (Figure 6E). The interaction between treatments and cultivars had a significant effect on adaxial trichome density (df = 3, *p* < 0.01, Figure 6F). When the average trichome density was compared across treatments and cultivars, the trend was similar to that of adaxial trichomes for the cultivar effect (df = 1, *p* < 0.05, Figure 6G), treatment effect (df = 3, *p* < 0.001, Figure 6H), and interaction (df = 3, *p* < 0.001, Figure 6I). Blackhawk had significantly more adaxial and total trichomes than Magellan immediately after the treatment, while there was no significant difference between the cultivars for abaxial density. This suggests that Blackhawk exhibits a stronger early response to herbivory and is subjected to greater changes under drought and herbivory stress conditions.

### 3.6 Trichome Density Six Days After the Treatment Period

**FIGURE 7:**
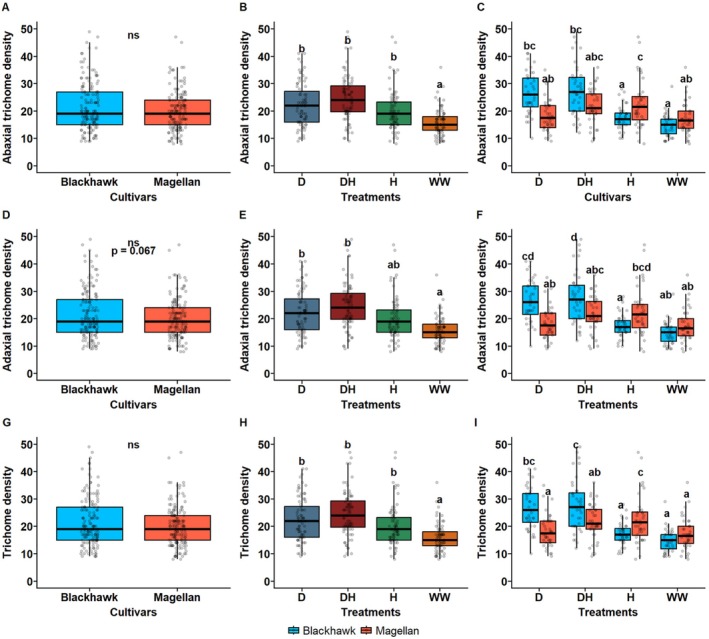
Effects of treatments and cultivars on leaf trichomes a week after ending the treatment. (A) Adaxial trichome density for two cultivars, (B) under drought and herbivory treatment, and (C) under interactive effects of treatments and cultivars. (D) Abaxial trichome density for two cultivars, (E) under drought and herbivory treatment, and (F) under interactive effects of treatments and cultivars. (G) Average trichome density (pooled across adaxial and abaxial surface) for two cultivars, (H) under drought and herbivory treatment, and (I) under interactive effects of treatments and cultivars. The leaves were excised on the same day treatment period ended. Leaf trichome density was measured by observing them under a compound microscope (0.086mm^2^ area) under 10× magnification. Drought and herbivory treatments are drought (D), drought × herbivory (DH), herbivory (H), and well‐watered (WW). Different letters in treatments indicate significant differences at the 5% level of significance, and data are presented as mean ± SE (standard error). ns, not significant.

The trichome density was significantly affected by the treatments but not due to the cultivars after 6 days of ending the drought and herbivory treatment. Abaxial trichomes were similar in both cultivars (df = 1, *p* = 0.38, Figure 7A), whereas plants exposed to drought × herbivory (DH) and drought (D) treatments had the highest abaxial trichome densities (df = 3, *p* < 0.01, Figure 7B) and well‐watered (WW) and herbivory (H) plants had the lowest. The average abaxial trichome density for DH treatment was around 25–27 trichomes per 0.086mm^2^ which was almost two times more than WW treatment (12–15 trichomes per 0.086mm^2^). Similarly, interactive effects of treatment and cultivars were also significant on abaxial trichomes, with Blackhawk having the highest density of trichomes under D and DH treatments (df = 3, *p* < 0.001). However, Magellan had significantly higher adaxial trichomes under herbivory compared to Blackhawk (Figure 7C).

Similarly, adaxial trichome density was not affected by cultivars (df = 1, *p* = 0.067, Figure 6D), but the treatment had significant effects; DH, D, and H treatment had higher trichomes compared to WW treatment (df = 3, *p* < 0.001, Figure 7E). The interaction effects between cultivars and treatments on adaxial trichome density followed a similar trend to abaxial trichomes (Figure 7F). When the average trichome density was compared across the cultivars, the cultivars did not affect the trichomes (df = 1, *p* = 0.596, Figure 7G), but the treatment effect was prominent and DH, D, and H treatment had higher trichomes compared to WW treatment (df = 3, *p* < 0.001, Figure 7H). The interaction between drought and herbivory treatment was significant and Blackhawk had the highest density of trichomes under D and DH treatments (df = 3, *p* < 0.001, Figure 7I). But Magellan had higher trichomes under herbivory compared to Blackhawk, which was also observed for adaxial trichomes (Figure 7I). The results reveal that the effects of drought and herbivory persist for a longer period even after the plants recovered from the stressed conditions and cultivar and stress treatment may interact with each other to strongly influence the trichome density of soybean leaves.

### 3.7 Comparison of Trichomes Immediately and a Week After Treatment Ending

The leaf trichome density was compared between 0 and 6 days (immediately and a week after ending the treatment respectively) and in interaction with the cultivars and treatments. The trichome density was significantly higher when examined a week after the treatment ended compared to immediately after the treatment period (df = 1, *p* = 0.013, Figure S4A). Under the interaction of cultivars and days after treatment, Magellan had higher trichomes at 6 days after the treatment ended (df = 2, *p* = 0.02, Figure S4B). The interaction between treatments and days after treatment showed significant effects, and WW treatment had the lowest trichomes while D and DH treatments had the highest trichomes during both measurements at 0 and 6 days after the treatment ended (df = 6, *p* < 0.001, Figure S4C). The three‐way interaction between days after treatment, cultivars and treatments had significant effects on the trichome density, and Magellan and Blackhawk both had higher trichomes on 0 and 6 days under DH treatments while the lowest was observed on both observations under WW treatment (df = 6, *p* < 0.001, Figure S4D). The results show that trichome induction due to drought and herbivory treatment persists for a longer period even after the plants have recovered from stressed conditions, and that cultivars play a critical role in driving the presence of trichomes in soybeans.

### 3.8 Fall Armyworm Mass Gain

**FIGURE 8:**
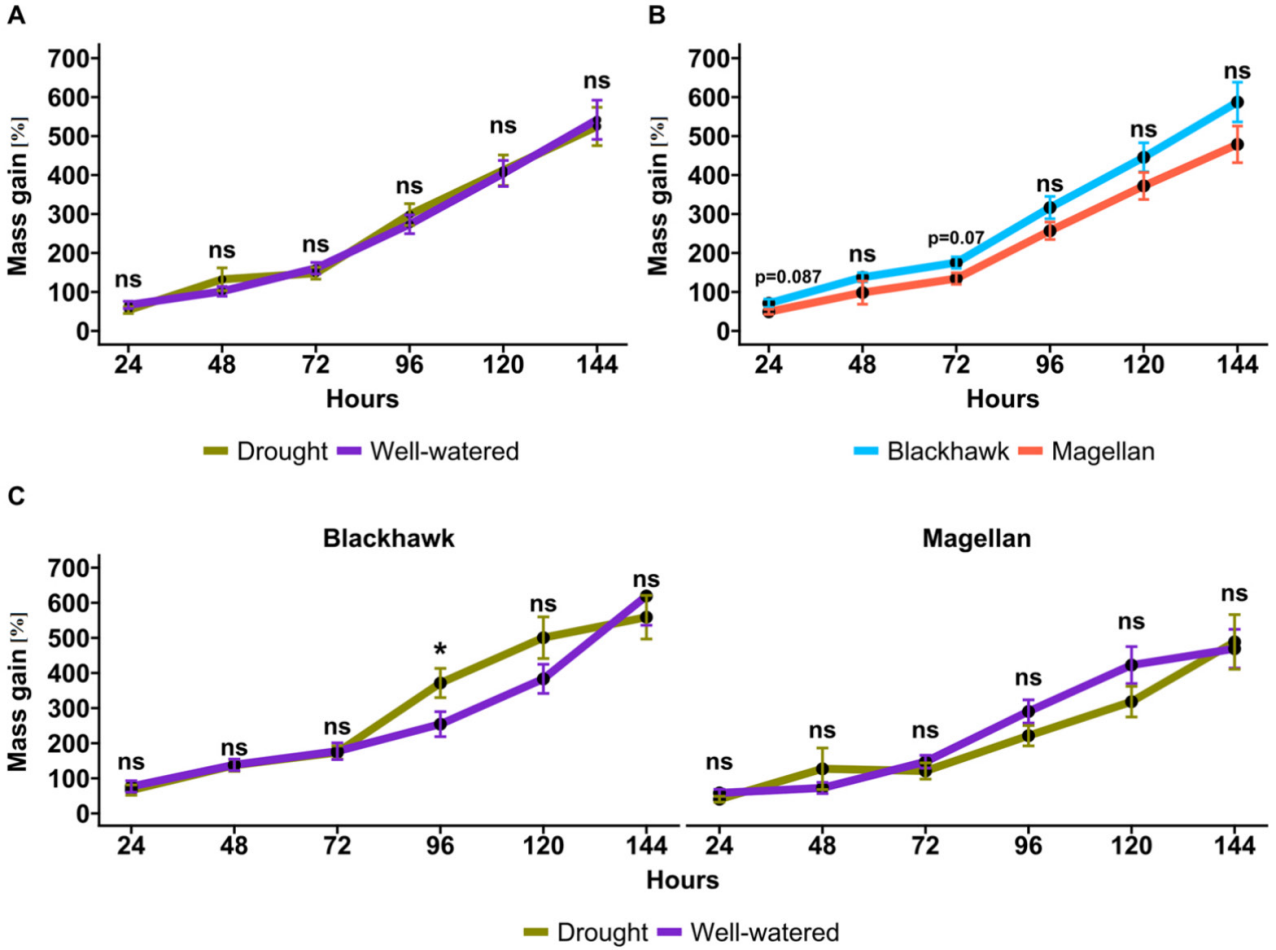
Mass gain (%) of fall armyworm (FAW) at 24, 48, 72, 96, 120, 144 h after putting them on the plants. FAW mass gain when they feed on (A) drought and well‐watered soybean plants, and (B) two cultivars (Blackhawk and Magellan) at 24, 48, 72, 96, 120, 144 h after putting them on the plants. (C) The interactive effects of treatment and cultivars on FAW mass gain at 24, 48, 72, 96, 120, 144 h after putting them on the plants. * *p* < 0.05; **p < 0.01; ***p < 0.001; ns, not significant.

Fall armyworm (FAW) mass gain was not significantly affected when they fed on drought stressed versus well‐watered plants (df = 3, *p* > 0.05, Figure 8A). Likewise, cultivars did not affect the mass gain of FAW as well (df = 3, *p* > 0.05). However, FAW gained more mass on Blackhawk than on Magellan throughout the observational period (24, 48, 72, 96, 120, and 144 h; Figure 8B). Interestingly, the interactive effects reveal that FAW gained significantly more mass in Blackhawk when they fed on drought stressed plants at 96 h after the FAW were released on the plants (df = 3, *p* = 0.02, Figure 8C). Although interaction between cultivars and drought treatment was not significant for other observations, contrasting trends were observed for mass gain between Blackhawk and Magellan from 72 to 120 h; FAW mass gain was higher in Blackhawk while lower in Magellan when they fed on drought stressed plants (Figure 8C). Although FAW mass gain remained unaffected despite feeding on drought stressed treatment, when analyzed in interaction with the cultivars, a trend was observed where higher drought tolerance in Magellan potentially led to lower mass gain of FAW compared to Blackhawk.

## 4 Discussion

This study presents findings from a comprehensive examination of soybean performance under concurrent drought and herbivory. The results from these experiments suggest that drought and herbivory interaction is also driven by the inherent tolerance of cultivars against stressors. Recently, Gautam et al. (2024) showed that drought followed by SBL herbivory caused overcompensation in physiological traits leading to synergistic negative effects on yield and fitness of soybeans. Our study examined the effects of concurrent drought and FAW herbivory simultaneously on soybean morpho‐physiological traits. Our findings show that drought and herbivory significantly affected the available water content, physiology and defense, and FAW growth in a cultivar dependent manner. More importantly, our results indicate that being a generalist herbivore, FAW can slightly benefit when fed on drought susceptible cultivars like Blackhawk, since they were able to gain more mass (although non‐significant during most of the observations) during the drought period. Additionally, we show that drought was the greatest driver of physiological change and had the most influence in reducing plant height, photosynthesis, stomatal conductance, and transpiration regardless of cultivar identity.

### 4.1 Soil Moisture Rapidly Declines Under Concurrent Drought and Herbivory Treatment

As expected, the volumetric soil water content rapidly declined under D and DH treatments, but H and WW treatments were similar, with H having slightly lower water content throughout the experiment (Figure 2). This suggested that although herbivory by FAW causes water loss due to increased transpiration (Kareem et al. 2022), it is not on par with the direct effect under drought stress. In terms of cultivar effects, Magellan had a higher soil water content and the rate of decline was also slower, which was expected due to its tolerance to drought compared to Blackhawk, which is drought susceptible (Sammons et al. 1978; He 2008; Cohen et al. 2021). This was evident from the interaction between cultivars and treatments (Figure 2C). Drought‐tolerant cultivars are able to retain soil moisture longer with better water use efficiency (Ayala et al. 2024) and thus minimize the impacts of water stress on their morpho‐physiological traits.

### 4.2 Concurrent Drought and Herbivory Treatments Negatively Affect Soybean Morpho‐Physiology

In the current study, plant height was significantly lower under D and DH treatments compared to H and WW, and Blackhawk was taller compared to Magellan (Figure 3A,B), which could be due to their variation in inherent growth rates. Although Sammons et al. (1978) stated that drought‐tolerant cultivars have better growth rates, our study shows that Blackhawk, having a higher growth rate leading to more height, may have resulted in increased water consumption as well. This is because the interactive effects of cultivars and treatments on plant height revealed that Blackhawk experienced more changes in plant height (reduced due to D and DH treatment), whereas Magellan remained more stable throughout the treatment period (Figure 3C). As such, our results imply that drought tolerance gives stability to cultivars in terms of growth rate when they are under concurrent drought and herbivory stress.

At the same time, leaf chlorophyll content was also affected by drought and herbivory treatments; DH treatment had the highest chlorophyll content, but H treatment had the lowest on the last two observations (4 and 8 DAT; Figure 4). Contrary to our findings, Makbul and Güler (2011) reported a significant reduction in leaf chlorophyll content under drought stress. Moreover, Blackhawk consistently had higher leaf chlorophyll content, but the interactive effects of treatment and cultivar were not significant. A recent study found that mild drought stress could lead to increased chlorophyll content which may decrease continuously under prolonged severe drought conditions. Such an increment in leaf chlorophyll content is often a measure adopted by the plants to protect their photosynthetic efficiency but a complete understanding of such mechanisms would require additional examination of chlorophyll fluorescence parameters (Yan et al. 2024).

More importantly, net photosynthesis rate, stomatal conductance, and transpiration rate rapidly declined under D and DH treatments after 2 DAT (Figure 5). This suggested that as the degree of drought intensified, the damage on the photosynthetic apparatus was more severe (Yan et al. 2024). Furthermore, the drought‐tolerant cultivar Magellan had significantly higher net photosynthesis than Blackhawk, but Blackhawk had higher stomatal conductance and transpiration rates, which clearly explains the cause for the accelerated decline in volumetric water content (Figure 5B,D,F). The treatments also interacted with cultivars, which revealed that Blackhawk experienced early decline while Magellan had a stable but gradual decline in physiological traits. This is mainly because drought tolerance traits of cultivars allow them to maintain the leaf photosynthetic ability for a prolonged period in response to declining soil moisture (Sammons et al. 1978; Wang et al. 2024) was evident from the trend observed in Magellan.

Interestingly, concurrent drought and herbivory did not show compensation effects on physiological traits as we observed in our previous experiments with SBL (Gautam et al. 2024). This could be due to the nature of DH stress imposed on the plants; previously drought was followed by a recovery phase and then herbivory was imposed whereas in the current experiment, continuous drought and herbivory stress were maintained concurrently to better mimic field conditions. Additionally, the use of FAW in the current study compared to SBL in previous experiments also could have led to the differing results. SBL, being more specialized to soybeans, could significantly differ in terms of their oral secretions compared to FAW, which is a generalist and does not prefer soybean as much as SBL. Interestingly, oral secretions can significantly impact stomatal regulation and thereby subsequent physiological traits of the plants too (Lin et al. 2022), which could be one of the major reasons for the discrepancy in our observations in the two experiments. Furthermore, the principal component analysis also revealed that D and DH treatments were more similar and spread out more compared to the H treatment, implying that D brought a stronger physiological response as compared to H by itself (Figure S2). Although such comparisons for drought and herbivory stress are uncommon across agricultural crops, seedling recovery has been found to be more dependent on past drought incidence rather than past herbivory infestation (Bansal et al. 2013), implying that drought is a dominant stressor compared to herbivory in plants. Not only herbivory, in fact, drought has been proven to predominate over other stressors such as heat stress. Zhou et al. (2017) found that drought predominantly affected tomato physiological responses causing the responses under heat stress to be very similar to what was observed under drought. In the same line, our observation also indicates a strong similarity between D and DH treatments which implies that drought is the dominant stressor under this concurrent drought and herbivory stress.

### 4.3 Concurrent Drought and Herbivory Positively Affect Trichome Density

As expected, higher trichomes were found under concurrent DH treatment compared to WW treatments for both adaxial and abaxial surfaces (Figure 6B,E). The average trichome density across both adaxial and abaxial surfaces was higher under DH and H treatments than WW treatments (Figure 6H). These results clearly suggest that trichome induction is an adopted mechanism by soybean plants under drought and herbivory stress, and such induction is strongly prominent when the plants experience both drought and herbivory stress concurrently. Recently, we showed that drought and herbivory by SBL in a sequence can also induce trichomes in soybeans (Gautam et al. 2026). There has been ample research on trichome‐mediated adaptation against stress in plants, primarily against abiotic and biotic stressors like drought and herbivory (Kaur and Kariyat 2020; Chen et al. 2022). In terms of cultivars, although Magellan was expected to induce more trichomes owing to its drought‐tolerant traits, Blackhawk was found to have higher trichome density even under the interactive effects of treatments and cultivars. These results suggest that fast‐growing cultivars like Blackhawk are less tolerant of stress but possess greater plasticity in terms of morphological parameters, while drought‐tolerant cultivars like Magellan may possess more prolonged and efficient defense strategies.

The observed effects for cultivars, however, changed a week after the treatment ended and the interactive effects of cultivars and treatments changed substantially. While other treatment effects remained the same, the induced trichome density under the H treatment was significantly higher under Magellan than Blackhawk. This result showed that along with being a drought tolerant cultivar, Magellan also possessed a strong response against herbivory, although it manifested at the later stages of plant growth. At the same time, the induced trichome density was found to be significantly greater when observed a week after the treatment ended as compared to immediately after the treatment ended. This clearly shows the prominent effects of concurrent drought and herbivory on leaf trichomes. Also, cultivar specific traits have a strong influence on trichome induction under drought and herbivory stress. More importantly, although drought is more dominant compared to herbivory, they can have additive effects on trichome density as observed from this study.

### 4.4 FAW Larval Feeding Is Unaffected by Drought Stress

Initially, we expected FAW to perform better since generalist herbivores have been found to feed more on drought stressed plants in contrast to specialized herbivores (Showler and Moran 2003; Gutbrodt et al. 2011). In agreement with the established notion, we also found that SBL preferred well‐watered plants and were least attracted to the drought‐stressed plants (Gautam et al. 2024). In line with our initial hypothesis, no significant effects of drought stress and cultivars were observed for FAW mass gain. However, the interaction between cultivar and treatments revealed that FAW mass gain followed an opposing trend in Blackhawk and Magellan when they fed on drought stressed plants (Figure 8C). FAW gained more mass on drought‐stressed plants in Blackhawk at 96 h after treatment. But in Magellan, FAW gained less mass on drought stressed plants and it was non‐significant throughout the experimental duration. This could be largely attributed to the drought tolerant traits of Magellan which not only relates to their better water use efficiency, but also their better physiological status. Another finding from this observation is that FAW, as a generalist herbivore, could be a problematic pest in drought susceptible cultivars like Blackhawk and can become a major crop pest in various abiotic stress conditions due to their adaptive and non‐selective feeding behavior. Utilization of plant nutrition by insect herbivores is another factor that has significant effects on herbivore performance. For instance, the tree locust *Anaeridium melanorhodon* is known to use specific phenolic compounds instead of phenolic amino acids for cuticle sclerotization while certain bruchid beetles can use canavanine, a toxic compound to many animals, that is found in seeds of many legume crops (Cockfield 1988). At the same time, drought stress also affects plant nitrogen and protein content at different stages of the plants, including seedling stage (Gautam and Kariyat 2025b). Thus, future studies should assess plant nutrition quality under both drought and herbivory and also include specialist and generalist herbivores for comparisons. However, since we used only two cultivars due to the logistical constraints of comprehensive data collection, with contrasting drought tolerance, further screening of a large number of genotypes could aid in extrapolating the direct application of this study in studying drought and herbivory susceptibility in soybeans. Moreover, the examination of chemical defenses in both drought susceptible and tolerant cultivars could provide a better understanding of defense mechanisms in soybeans under drought and herbivory interactions. Results from our previous experiments combined with the current findings provide substantial validation to the genotype‐based inferences drawn in this study. Future experiments should also explore the changes in leaf metabolites and phytohormones under drought stress and the cascading effects on other life stages of FAW, including their reproductive abilities and fecundity traits to accurately inform about FAW performance under drought stress conditions in soybeans.

## 5 Conclusions

This study investigated the effects of concurrent drought and herbivory using two soybean cultivars of contrasting tolerance towards drought stress. The results revealed that concurrent drought and herbivory cause a significant decline in water content, and thereby physiological traits. However, it is the same for drought stress alone, and when studied independently, herbivory had little effect on water content compared to the well‐watered treatment. The negative effects are reflected in terms of reduced growth rates of plants, but the plants also experienced an increase in trichomes adapted for defense against abiotic and biotic stressors. We clearly show that concurrent drought and herbivory are very distinct compared to well‐watered treatments, but the effect is primarily driven by drought stress as most of the traits were similar with independent drought stress. Therefore, based on the results of this study, drought can be considered (or reconfirmed) as the predominant stressor compared to herbivory alone, since DH treatment resembled more closely drought stress rather than herbivory. On the other hand, FAW, as a generalist herbivore, is not negatively affected by drought stress; rather, it may even do better with drought susceptible cultivars. It will be an interesting avenue to test whether both sucking and chewing herbivores would perform similarly in soybeans under such stress conditions. Furthermore, since drought tolerance traits greatly influence how such morpho‐physiological traits are affected under concurrent drought and herbivory, future experiments should also prioritize the development of resilient cultivars targeted for stable yield and fitness under such conditions. These findings contribute to research emphasizing the need to consider both abiotic and biotic stress interactions when selecting the crops for their stress resilience.

## Author Contributions

R.K. and M.G. conceptualized the study. M.G. and A.P. carried out experiments and collected data. All the statistical analyses were performed by M.G. M.G. and A.P. wrote the first draft and R.K. and A.P. edited the draft and further versions of the draft. All the authors contributed to revising the manuscript and gave final approval for submission.

## Funding

This work was supported by start‐up funds of AFLS honors college.

## Conflicts of Interest

The authors declare no conflicts of interest.

## Supporting information


**Figure S1:** Estimation of trichome density in soybean leaves. (A) Non‐glandular trichomes present in soybean leaves. (B) Using a compound microscope at 10× magnification to observe the soybean leaf trichomes. The leaf disc was obtained by punching a hole across the leaf margin by avoiding the mid veins and trichomes were counted per 0.086 mm^2^ leaf area to obtain trichome density.
**Figure S2:** Principal component analysis (PCA) and interactive effects of treatments and cultivars for physiological traits of soybean. (A) PCA for physiological traits based on drought and herbivory treatments, and (B) based on cultivars. ‘Photo’ ‐ net photosynthesis rate, ‘Trans’ ‐ transpiration rate, ‘Cond’ – stomatal conductance, ‘Ci’ – intercellular CO_2_. Drought and herbivory treatments are drought (D), drought × herbivory (DH), herbivory (H), and well‐watered (WW). * *p* < 0.05; **p < 0.01; ***p < 0.001; ns, not significant.
**Figure S3:** Interactive effects of treatments and cultivars on physiological traits at different days after treatment (DAT). Treatment and cultivar interaction effects on (A) net photosynthesis rate, (B) stomatal conductance, and (C) transpiration rate. Drought and herbivory treatments are drought (D), drought × herbivory (DH), herbivory (H), and well‐watered (WW). * *p* < 0.05; **p < 0.01; ***p < 0.001; ns, not significant.
**Figure S4:** Comparison of trichomes across 0 and 6 days (a week including the day of ending the treatment) after the treatment period. treatments and cultivars on leaf trichomes a week after ending the treatment. (A) Average trichome density between 0 and 6 days after ending treatment, (B) under the interaction of cultivars and days after treatment, (C) under the interaction of treatment and days after treatment, and (D) under the interaction effects of treatment, cultivar, and days after ending treatment. The leaves were excised 0 days, and 6 days after the treatment ended. Leaf trichome density was measured by observing them under a compound microscope (0.086 mm^2^ area) under 10× magnification. Drought and herbivory treatments are drought (D), drought × herbivory (DH), herbivory (H), and well‐watered (WW). Different letters in treatments indicate significant differences at the 5% level of significance, and data are presented as mean ± SE (standard error). ns: not significant.
**Table S1:** Two different soybean cultivars used in the study.
**Table S2:** Full factorial analyses of the effects of concurrent drought and herbivory treatments and cultivars on volumetric soil water content.
**Table S3:** Full factorial analyses of the effects of concurrent drought and herbivory treatments and cultivars on soybean morpho‐physiological traits.
**Table S4:** Full factorial analyses of the effects of concurrent drought and herbivory treatments and cultivars on soybean leaf trichomes.
**Table S5:** Full factorial analyses of the effects of drought on FAW larval mass gain.

## Data Availability

The raw data collected and used in this manuscript will be made available on reasonable request to the corresponding author.
