# Supplementary material for: Under Concurrent Drought and Herbivory, Drought Dominates Herbivory on Morpho‐Physiological Responses in Soybean
Source: Physiol Plant. 2026 Jan 25;178(1):e70748. doi: 10.1111/ppl.70748 (PMC12832602; doi:10.1111/ppl.70748)
Supplement: Supplementary file 1 — Figure S1: Estimation of trichome density in soybean leaves. (A) Non‐glandular trichomes present in soybean leaves. (B) Using a compound microscope at 10× magnification to observe the soybean leaf trichomes. The leaf disc was obtained by punching a hole across the leaf margin by avoiding the mid veins and trichomes were counted per 0.086 mm2 leaf area to obtain trichome density. Figure S2: Principal component analysis (PCA) and interactive effects of treatments and cultivars for physiological traits of soybean. (A) PCA for physiological traits based on drought and herbivory treatments, and (B) based on cultivars. ‘Photo’ ‐ net photosynthesis rate, ‘Trans’ ‐ transpiration rate, ‘Cond’ – stomatal conductance, ‘Ci’ – intercellular CO2. Drought and herbivory treatments are drought (D), drought × herbivory (DH), herbivory (H), and well‐watered (WW). * p < 0.05; **p < 0.01; ***p < 0.001; ns, not significant. Figure S3: Interactive effects of treatments and cultivars on physiological traits at different days after treatment (DAT). Treatment and cultivar interaction effects on (A) net photosynthesis rate, (B) stomatal conductance, and (C) transpiration rate. Drought and herbivory treatments are drought (D), drought × herbivory (DH), herbivory (H), and well‐watered (WW). * p < 0.05; **p < 0.01; ***p < 0.001; ns, not significant. Figure S4: Comparison of trichomes across 0 and 6 days (a week including the day of ending the treatment) after the treatment period. treatments and cultivars on leaf trichomes a week after ending the treatment. (A) Average trichome density between 0 and 6 days after ending treatment, (B) under the interaction of cultivars and days after treatment, (C) under the interaction of treatment and days after treatment, and (D) under the interaction effects of treatment, cultivar, and days after ending treatment. The leaves were excised 0 days, and 6 days after the treatment ended. Leaf trichome density was measured by observing them under a compound microsc [file PPL-178-e70748-s001.docx]

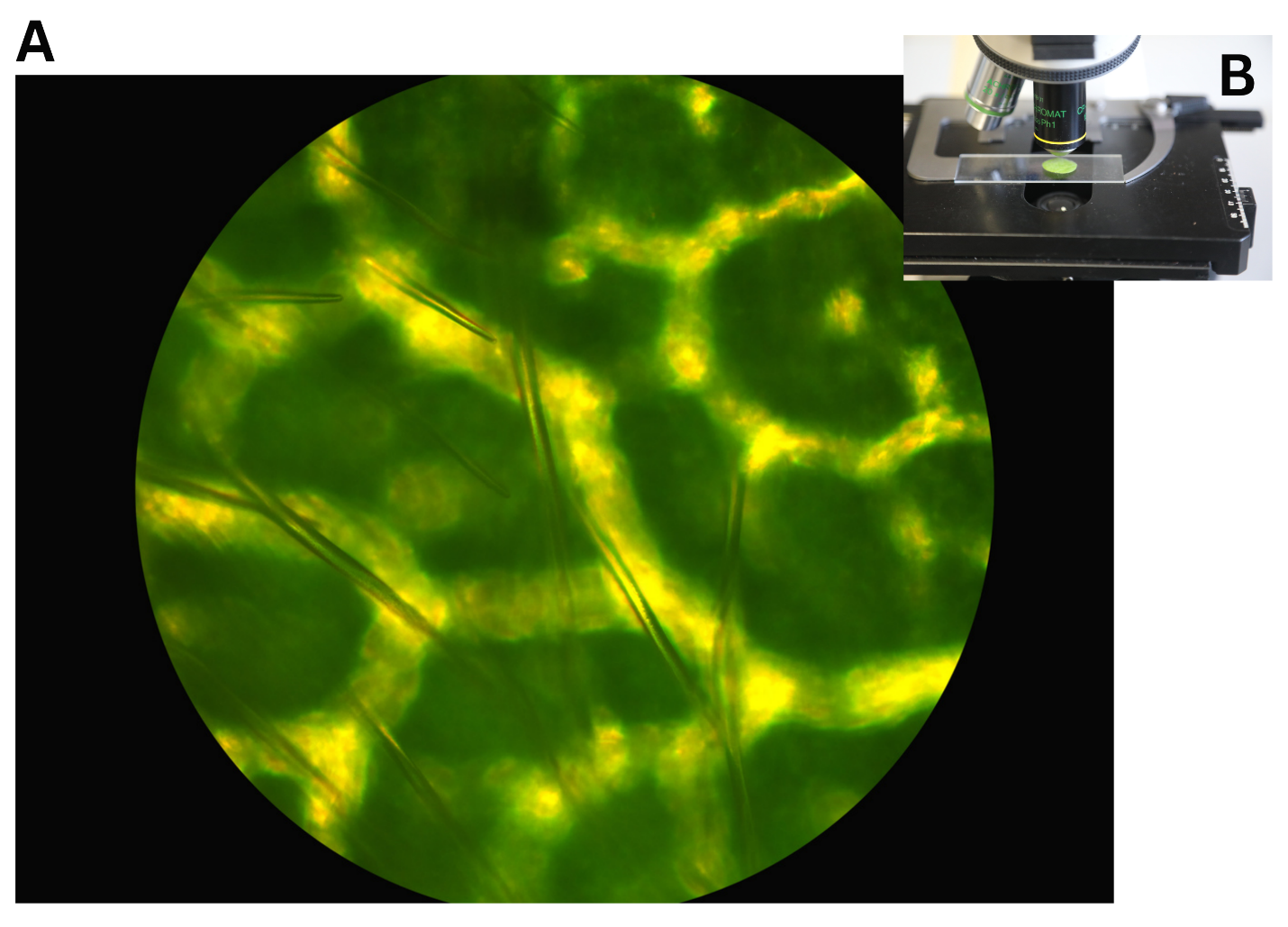


**Figure S1.** Estimation of trichome density in soybean leaves. (A) Non-glandular trichomes present in soybean leaves. (B) Using a compound microscope at 10× magnification to observe the soybean leaf trichomes. The leaf disc was obtained by punching a hole across the leaf margin by avoiding the mid veins and trichomes were counted per 0.086 mm^2^ leaf area to obtain trichome density.


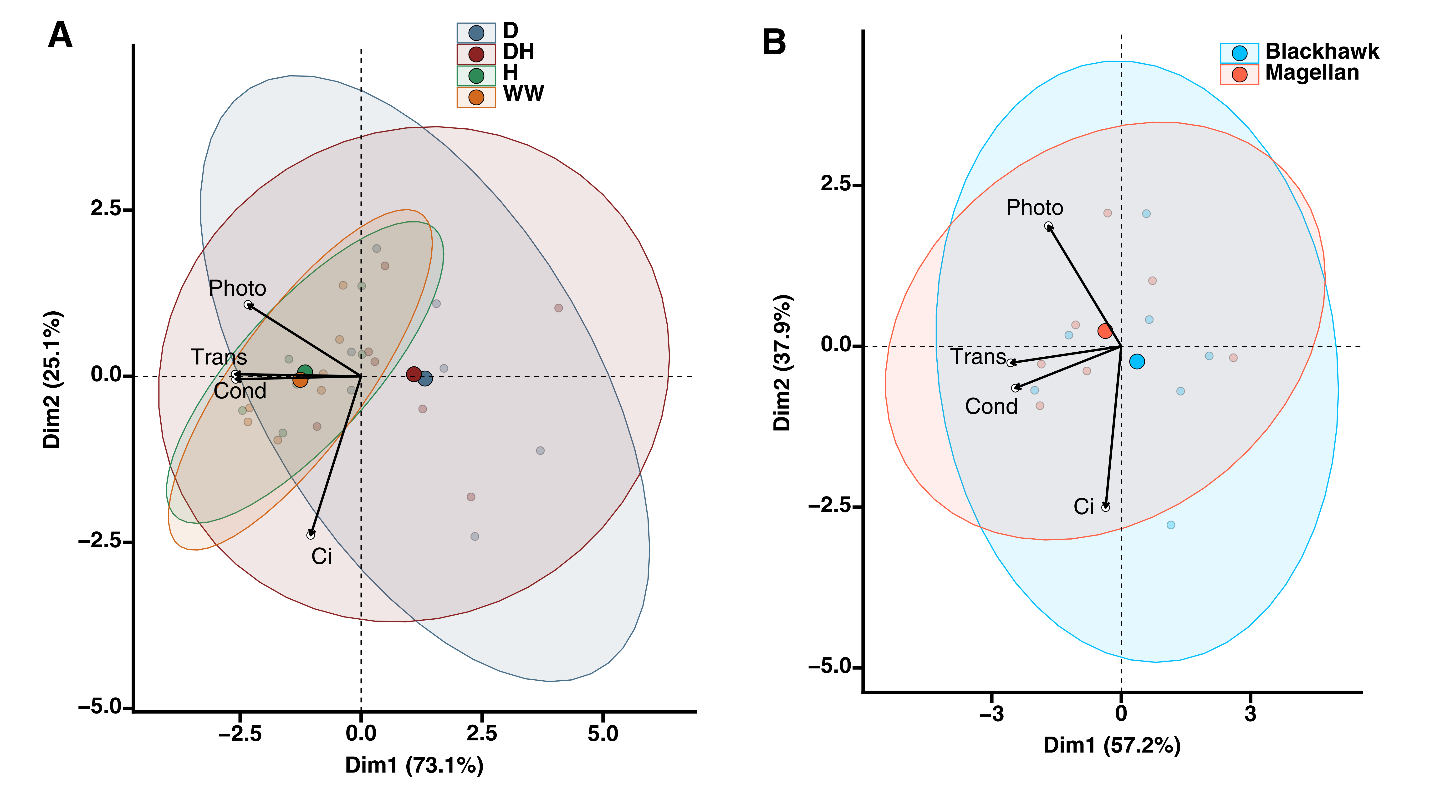
**Figure S2.** Principal component analysis (PCA) and interactive effects of treatments and cultivars for physiological traits of soybean. (A) PCA for physiological traits based on drought and herbivory treatments, and (B) based on cultivars. ‘Photo’ - net photosynthesis rate, ‘Trans’ - transpiration rate, ‘Cond’ – stomatal conductance, ‘Ci’ – intercellular CO_2_. Drought and herbivory treatments are drought (D), drought x herbivory (DH), herbivory (H), and well-watered (WW). * P < 0.05; **P < 0.01; ***P < 0.001; ns, not significant.


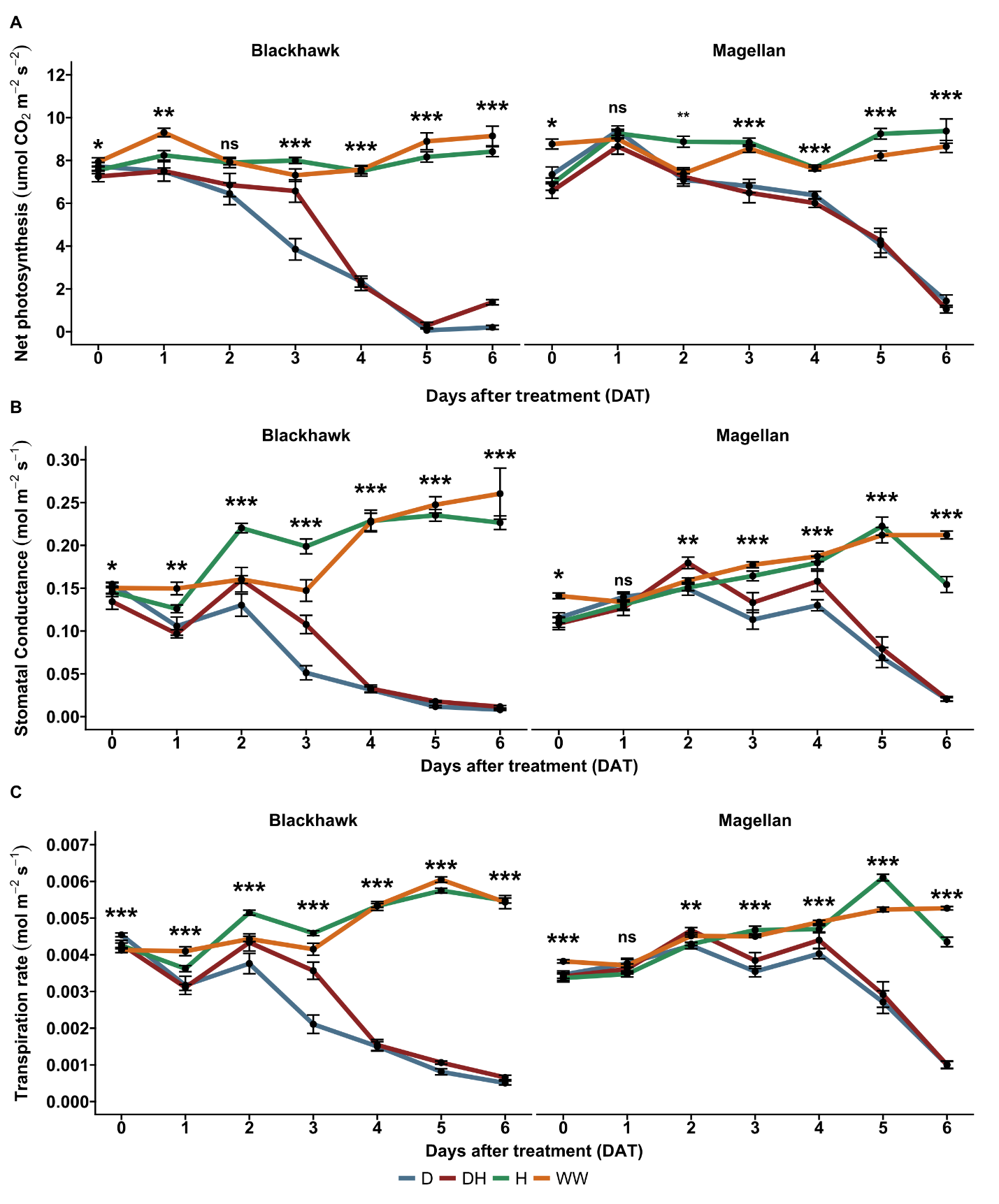
**Figure S3.** Interactive effects of treatments and cultivars on physiological traits at different days after treatment (DAT). Treatment and cultivar interaction effects on (A) net photosynthesis rate, (B) stomatal conductance, and (C) transpiration rate. Drought and herbivory treatments are drought (D), drought x herbivory (DH), herbivory (H), and well-watered (WW). * P < 0.05; **P < 0.01; ***P < 0.001; ns, not significant.


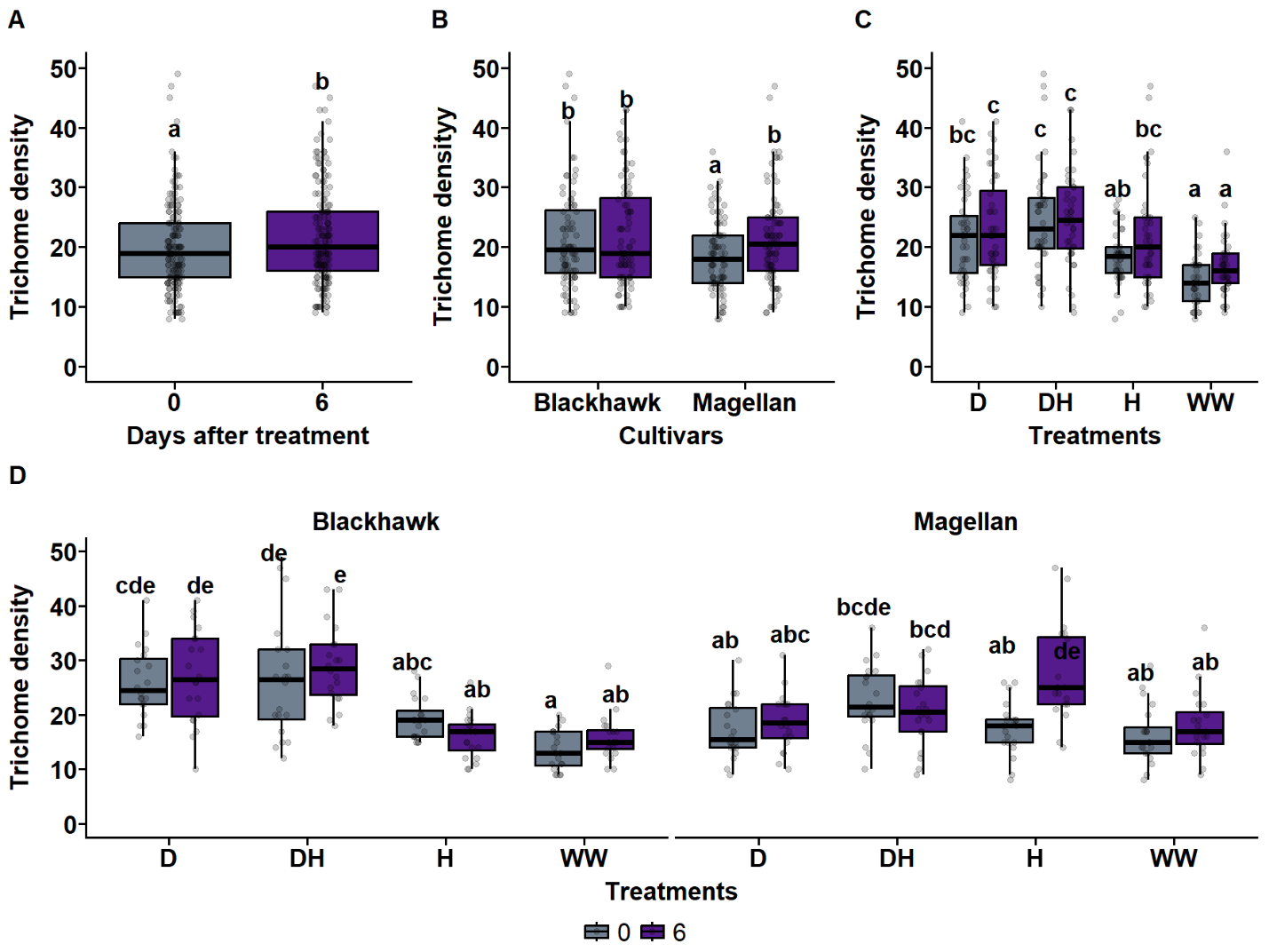
**Figure S4.** Comparison of trichomes across 0 and 6 days (a week including the day of ending the treatment) after the treatment period. treatments and cultivars on leaf trichomes a week after ending the treatment. (A) Average trichome density between 0 and 6 days after ending treatment, (B) under the interaction of cultivars and days after treatment, (C) under the interaction of treatment and days after treatment, and (D) under the interaction effects of treatment, cultivar, and days after ending treatment. The leaves were excised 0 days, and 6 days after the treatment ended. Leaf trichome density was measured by observing them under a compound microscope (0.086mm^2^ area) under 10X magnification. Drought and herbivory treatments are drought (D), drought x herbivory (DH), herbivory (H), and well-watered (WW). Different letters in treatments indicate significant differences at the 5 % level of significance, and data are presented as mean ± SE (standard error). ns: not significant.

**Table S1** Two different soybean cultivars used in the study

| ***Cultivar*** | ***Pedigree*** | ***Maturity group*** | ***Drought tolerance*** | ***Origin/ Developed*** | ***Reference*** |
| --- | --- | --- | --- | --- | --- |
| Blackhawk  (PI 584516) | Mukden x Richland | I | Susceptible | Iowa, United States | Sammons et al., (1978) |
| Magellan (PI 595362) | Sherman x Harper | IV | Tolerant | Kansas, United States | He (2008) |

All the cultivars are available at the National Laboratory for Genetic Resources Preservation. <https://npgsweb.ars-grin.gov/gringlobal/search>. This table is adapted from Table S1 in Gautam et al., (2024).

**Table S2** Full factorial analyses of the effects of concurrent drought and herbivory treatments and cultivars on volumetric soil water content

| **Variables** | **Source of variation** | **df** | **p value** |
| --- | --- | --- | --- |
| **Effect on Volumetric soil water content** | | | |
| **25 days after transplanting (25 DAT)** | | | |
| Volumetric soil water content | Treatments | 3 | 0.002** |
| Volumetric soil water content | Cultivar | 1 | 0.00338** |
| Volumetric soil water content | Treatments: Cultivars | 3 | 0.316 |
| **26 days after transplanting (26 DAT)** | | | |
| Volumetric soil water content | Treatments | 3 | <0.001*** |
| Volumetric soil water content | Cultivar | 1 | 0.019* |
| Volumetric soil water content | Treatments: Cultivars | 3 | 0.0625 |
| **27 days after transplanting (27 DAT)** | | | |
| Volumetric soil water content | Treatments | 3 | <0.001*** |
| Volumetric soil water content | Cultivar | 1 | 0.0445* |
| Volumetric soil water content | Treatments: Cultivars | 3 | <0.001*** |
| **28 days after transplanting (28 DAT)** | | | |
| Volumetric soil water content | Treatments | 3 | <0.001*** |
| Volumetric soil water content | Cultivar | 1 | 0.0027** |
| Volumetric soil water content | Treatments: Cultivars | 3 | 0.158 |
| **29 days after transplanting (29 DAT)** | | | |
| Volumetric soil water content | Treatments | 3 | <0.001*** |
| Volumetric soil water content | Cultivar | 1 | 0.00216** |
| Volumetric soil water content | Treatments: Cultivars | 3 | 0.2295 |
| **30 days after transplanting (30 DAT)** | | | |
| Volumetric soil water content | Treatments | 3 | <0.001*** |
| Volumetric soil water content | Cultivar | 1 | <0.001*** |
| Volumetric soil water content | Treatments: Cultivars | 3 | 0.708 |
| **31 days after transplanting (31 DAT)** | | | |
| Volumetric soil water content | Treatments | 3 | <0.001*** |
| Volumetric soil water content | Cultivar | 1 | <0.001*** |
| Volumetric soil water content | Treatments: Cultivars | 3 | 0.44 |

The above table is obtained from ANOVA followed by Tukey’s HSD post-hoc test. Treatments represent drought (D), concurrent drought and herbivory (DH), herbivory (H), and well-watered (WW) treatments. *, P < 0.05; **, P < 0.01; ***, P < 0.001.

**Table S3** Full factorial analyses of the effects of concurrent drought and herbivory treatments and cultivars on soybean morpho-physiological traits

| **Variables** | **Source of variation** | **df** | **p value** |
| --- | --- | --- | --- |
| **Effect on plant height** | | | |
| **0 days after treatment (0 DAT)** | | | |
| Plant height | Treatments | 3 | 0.6996 |
| Plant height | Cultivar | 1 | <0.001*** |
| Plant height | Treatments: Cultivars | 3 | 0.0548 |
| **2 days after treatment (2 DAT)** | | | |
| Plant height | Treatments | 3 | 0.209 |
| Plant height | Cultivar | 1 | <0.001*** |
| Plant height | Treatments: Cultivars | 3 | 0.0201* |
| **4 days after treatment (4 DAT)** | | | |
| Plant height | Treatments | 3 | 0.0415* |
| Plant height | Cultivar | 1 | <0.001*** |
| Plant height | Treatments: Cultivars | 3 | 0.0433* |
| **6 days after treatment (6 DAT)** | | | |
| Plant height | Treatments | 3 | <0.001*** |
| Plant height | Cultivar | 1 | <0.001*** |
| Plant height | Treatments: Cultivars | 3 | 0.00217** |
| **Effect on leaf chlorophyll content** | | | |
| **0 days after treatment (0 DAT)** |  |  |  |
| Leaf chlorophyll content | Treatments | 3 | 0.506 |
| Leaf chlorophyll content | Cultivar | 1 | <0.001*** |
| Leaf chlorophyll content | Treatments: Cultivars | 3 | 0.412 |
| **2 days after treatment (2 DAT)** |  |  |  |
| Leaf chlorophyll content | Treatments | 3 | 0.469 |
| Leaf chlorophyll content | Cultivar | 1 | 0.00411** |
| Leaf chlorophyll content | Treatments: Cultivars | 3 | 0.28601 |
| **4 days after treatment (4 DAT)** |  |  |  |
| Leaf chlorophyll content | Treatments | 3 | 0.0267* |
| Leaf chlorophyll content | Cultivar | 1 | <0.001*** |
| Leaf chlorophyll content | Treatments: Cultivars | 3 | 0.1548 |
| **6 days after treatment (6 DAT)** |  |  |  |
| Leaf chlorophyll content | Treatments | 3 | <0.001*** |
| Leaf chlorophyll content | Cultivar | 1 | <0.001*** |
| Leaf chlorophyll content | Treatments: Cultivars | 3 | 0.775 |
|  |  |  |  |
| **Effect on net photosynthesis rate** | | | |
| **0 days after treatment (0 DAT)** | | | |
| Net photosynthesis rate | Treatments | 3 | <0.001*** |
| Net photosynthesis rate | Cultivar | 1 | 0.2335 |
| Net photosynthesis rate | Treatments: Cultivars | 3 | 0.0112* |
| **1 days after treatment (1 DAT)** | | | |
| Net photosynthesis rate | Treatments | 3 | 0.00561** |
| Net photosynthesis rate | Cultivar | 1 | <0.001*** |
| Net photosynthesis rate | Treatments: Cultivars | 3 | 0.00259** |
| **2 days after treatment (1 DAT)** | | | |
| Net photosynthesis rate | Treatments | 3 | <0.001*** |
| Net photosynthesis rate | Cultivar | 1 | 0.14 |
| Net photosynthesis rate | Treatments: Cultivars | 3 | 0.156 |
| **3 days after treatment (3 DAT)** | | | |
| Net photosynthesis rate | Treatments | 3 | <0.001*** |
| Net photosynthesis rate | Cultivar | 1 | <0.001*** |
| Net photosynthesis rate | Treatments: Cultivars | 3 | <0.001*** |
| **4 days after treatment (4 DAT)** | | | |
| Net photosynthesis rate | Treatments | 3 | <0.001*** |
| Net photosynthesis rate | Cultivar | 1 | <0.001*** |
| Net photosynthesis rate | Treatments: Cultivars | 3 | <0.001*** |
| **5 days after treatment (5 DAT)** | | | |
| Net photosynthesis rate | Treatments | 3 | <0.001*** |
| Net photosynthesis rate | Cultivar | 1 | <0.001*** |
| Net photosynthesis rate | Treatments: Cultivars | 3 | <0.001*** |
| **6 days after treatment (6 DAT)** | | | |
| Net photosynthesis rate | Treatments | 3 | <0.001*** |
| Net photosynthesis rate | Cultivar | 1 | 0.1101 |
| Net photosynthesis rate | Treatments: Cultivars | 3 | 0.0101* |
|  |  |  |  |
| **Effect on transpiration rate** | | | |
| **0 days after treatment (0 DAT)** | | | |
| Transpiration rate | Treatments | 3 | 0.0516 |
| Transpiration rate | Cultivar | 1 | <0.001*** |
| Transpiration rate | Treatments: Cultivars | 3 | <0.001*** |
| **1 days after treatment (1 DAT)** | | | |
| Transpiration rate | Treatments | 3 | 0.0215* |
| Transpiration rate | Cultivar | 1 | 0.1232 |
| Transpiration rate | Treatments: Cultivars | 3 | 0.00265** |
| **2 days after treatment (1 DAT)** | | | |
| Transpiration rate | Treatments | 3 | <0.001*** |
| Transpiration rate | Cultivar | 1 | 0.896 |
| Transpiration rate | Treatments: Cultivars | 3 | <0.001*** |
| **3 days after treatment (3 DAT)** | | | |
| Transpiration rate | Treatments | 3 | <0.001*** |
| Transpiration rate | Cultivar | 1 | <0.001*** |
| Transpiration rate | Treatments: Cultivars | 3 | <0.001*** |
| **4 days after treatment (4 DAT)** | | | |
| Transpiration rate | Treatments | 3 | <0.001*** |
| Transpiration rate | Cultivar | 1 | <0.001*** |
| Transpiration rate | Treatments: Cultivars | 3 | <0.001*** |
| **5 days after treatment (5 DAT)** | | | |
| Transpiration rate | Treatments | 3 | <0.001*** |
| Transpiration rate | Cultivar | 1 | <0.001*** |
| Transpiration rate | Treatments: Cultivars | 3 | <0.001*** |
| **6 days after treatment (6 DAT)** | | | |
| Transpiration rate | Treatments | 3 | <0.001*** |
| Transpiration rate | Cultivar | 1 | 0.14 |
| Transpiration rate | Treatments: Cultivars | 3 | <0.001*** |
|  |  |  |  |
| **Effect on stomatal conductance** | | | |
| **0 days after treatment (0 DAT)** | | | |
| Stomatal conductance | Treatments | 3 | <0.001*** |
| Stomatal conductance | Cultivar | 1 | <0.001*** |
| Stomatal conductance | Treatments: Cultivars | 3 | 0.0253* |
| **1 days after treatment (1 DAT)** | | | |
| Stomatal conductance | Treatments | 3 | 0.00129** |
| Stomatal conductance | Cultivar | 1 | 0.0129* |
| Stomatal conductance | Treatments: Cultivars | 3 | 0.00286** |
| **2 days after treatment (1 DAT)** | | | |
| Stomatal conductance | Treatments | 3 | <0.001*** |
| Stomatal conductance | Cultivar | 1 | 0.177 |
| Stomatal conductance | Treatments: Cultivars | 3 | <0.001*** |
| **3 days after treatment (3 DAT)** | | | |
| Stomatal conductance | Treatments | 3 | <0.001*** |
| Stomatal conductance | Cultivar | 1 | 0.00241** |
| Stomatal conductance | Treatments: Cultivars | 3 | <0.001*** |
| **4 days after treatment (4 DAT)** | | | |
| Stomatal conductance | Treatments | 3 | <0.001*** |
| Stomatal conductance | Cultivar | 1 | <0.001*** |
| Stomatal conductance | Treatments: Cultivars | 3 | <0.001*** |
| **5 days after treatment (5 DAT)** | | | |
| Stomatal conductance | Treatments | 3 | <0.001*** |
| Stomatal conductance | Cultivar | 1 | <0.001*** |
| Stomatal conductance | Treatments: Cultivars | 3 | <0.001*** |
| **6 days after treatment (6 DAT)** | | | |
| Stomatal conductance | Treatments | 3 | <0.001*** |
| Stomatal conductance | Cultivar | 1 | 0.0031** |
| Stomatal conductance | Treatments: Cultivars | 3 | <0.001*** |

The above table is obtained from ANOVA followed by Tukey’s HSD post-hoc test. Treatments represent drought (D), concurrent drought and herbivory (DH), herbivory (H), and well-watered (WW) treatments. *, P < 0.05; **, P < 0.01; ***, P < 0.001.

**Table S4** Full factorial analyses of the effects of concurrent drought and herbivory treatments and cultivars on soybean leaf trichomes

| **Variables** | **Source of variation** | **df** | **p value** |
| --- | --- | --- | --- |
| **Effect on trichomes immediately after ending treatments** | | | |
| Adaxial trichomes | Treatments | 3 | <0.001*** |
| Adaxial trichomes | Cultivar | 1 | 0.00435** |
| Adaxial trichomes | Treatments: Cultivars | 3 | 0.00597** |
| Abaxial trichomes | Treatments | 3 | <0.001*** |
| Abaxial trichomes | Cultivar | 1 | 0.2397 |
| Abaxial trichomes | Treatments: Cultivars | 3 | <0.001*** |
| Average trichomes | Treatments | 3 | <0.001*** |
| Average trichomes | Cultivar | 1 | 0.0135* |
| Average trichomes | Treatments: Cultivars | 3 | <0.001*** |
| **Effect on trichomes a week after ending treatments** | | | |
| Adaxial trichomes | Treatments | 3 | <0.001*** |
| Adaxial trichomes | Cultivar | 1 | 0.067 |
| Adaxial trichomes | Treatments: Cultivars | 3 | <0.001*** |
| Abaxial trichomes | Treatments | 3 | 0.00184** |
| Abaxial trichomes | Cultivar | 1 | 0.3804 |
| Abaxial trichomes | Treatments: Cultivars | 3 | <0.001*** |
| Average trichomes | Treatments | 3 | <0.001*** |
| Average trichomes | Cultivar | 1 | 0.596 |
| Average trichomes | Treatments: Cultivars | 3 | <0.001*** |
| **Comparison of trichomes (0 versus 6 days after treatment ended (DAT))** | | | |
| Average trichomes | Days | 1 | 0.0128* |
| Average trichomes | Days: Cultivar | 2 | 0.0208* |
| Average trichomes | Days: Treatment | 6 | <0.001*** |
| Average trichomes | Days: Cultivar: Treatment | 6 | <0.001*** |

The above table is obtained from ANOVA followed by Tukey’s HSD post-hoc test. Treatments represent drought (D), concurrent drought and herbivory (DH), herbivory (H), and well-watered (WW) treatments. *, P < 0.05; **, P < 0.01; ***, P < 0.001.

**Table S5** Full factorial analyses of the effects of drought on FAW larval mass gain

| **Variables** | **Source of variation** | **df** | **p value** |
| --- | --- | --- | --- |
| **Effects of drought stress on FAW mass gain** | | | |
| 24 hours mass gain | Cultivar | 1 | 0.309 |
| 24 hours mass gain | Treatments: Cultivars | 3 | 0.087 |
| 24 hours mass gain | Treatments | 3 | 0.768 |
| 48 hours mass gain | Cultivar | 1 | 0.372 |
| 48 hours mass gain | Treatments: Cultivars | 3 | 0.292 |
| 48 hours mass gain | Treatments | 3 | 0.421 |
| 72 hours mass gain | Cultivar | 1 | 0.572 |
| 72 hours mass gain | Treatments: Cultivars | 3 | 0.0708 |
| 72 hours mass gain | Treatments | 3 | 0.6154 |
| 96 hours mass gain | Cultivar | 1 | 0.5209 |
| 96 hours mass gain | Treatments: Cultivars | 3 | 0.0202* |
| 96 hours mass gain | Treatments | 3 | 0.1395 |
| 120 hours mass gain | Cultivar | 1 | 0.8858 |
| 120 hours mass gain | Treatments: Cultivars | 3 | 0.1920 |
| 120 hours mass gain | Treatments | 3 | 0.0509 |
| 144 hours mass gain | Cultivar | 1 | 0.829 |
| 144 hours mass gain | Treatments: Cultivars | 3 | 0.17 |
| 144 hours mass gain | Treatments | 3 | 0.613 |

The above table is obtained from ANOVA followed by Tukey’s HSD post-hoc test. Treatments represent drought (D), concurrent drought and herbivory (DH), herbivory (H), and well-watered (WW) treatments. *, P < 0.05; **, P < 0.01; ***, P < 0.001.
